# *De novo* assembly of a chromosome-scale reference genome for the northern flicker *Colaptes auratus*

**DOI:** 10.1093/g3journal/jkaa026

**Published:** 2020-12-09

**Authors:** Jack P Hruska, Joseph D Manthey

**Affiliations:** Department of Biological Sciences, Texas Tech University, Lubbock, TX 79409-43131, USA

**Keywords:** *Colaptes auratus*, woodpeckers, PacBio, Hi-C, genome assembly

## Abstract

The northern flicker, *Colaptes auratus*, is a widely distributed North American woodpecker and a long-standing focal species for the study of ecology, behavior, phenotypic differentiation, and hybridization. We present here a highly contiguous *de novo* genome assembly of *C. auratus*, the first such assembly for the species and the first published chromosome-level assembly for woodpeckers (Picidae). The assembly was generated using a combination of short-read Chromium 10× and long-read PacBio sequencing, and further scaffolded with chromatin conformation capture (Hi-C) reads. The resulting genome assembly is 1.378 Gb in size, with a scaffold N50 of 11  and a scaffold L50 of 43.948 Mb. This assembly contains 87.4–91.7% of genes present across four sets of universal single-copy orthologs found in tetrapods and birds. We annotated the assembly both for genes and repetitive content, identifying 18,745 genes and a prevalence of ∼28.0% repetitive elements. Lastly, we used fourfold degenerate sites from neutrally evolving genes to estimate a mutation rate for *C. auratus*, which we estimated to be 4.007 × 10^−9^ substitutions/site/year, about 1.5× times faster than an earlier mutation rate estimate of the family. The highly contiguous assembly and annotations we report will serve as a resource for future studies on the genomics of *C. auratus* and comparative evolution of woodpeckers.

## Introduction

The northern flicker *Colaptes auratus* is a polytypic North American woodpecker with a distribution spanning from Alaska to northern Nicaragua, Cuba, and the Cayman Islands. *Colaptes auratus* consists of up to 13 described subspecies (Gill *et al.* 2020) and 5 morphological groups (Short 1982). Currently, the taxonomy of *C. auratus* is uncertain; some authorities consider it to form a species complex along with the gilded flicker *Colaptes chrysoides*, while others have suggested that one of the subspecies, *C.auratus mexicanoides*, is best considered a separate species (del Hoyo *et al.* 2014). In addition, hybridization between morphological groups in secondary contact is prevalent, primarily between the yellow-shafted and red-shafted flickers, who form a hybrid zone that extends from northern Texas to southern Alaska (Wiebe and Moore 2020). The yellow-shafted/red-shafted hybrid zone has become a prominent study system for the consequences of secondary contact (*e.g.*, Moore and Koenig 1986; Wiebe 2000). Despite there being marked phenotypic differentiation between red-shafted and yellow-shafted flickers, genetic divergence between these groups is remarkably shallow, even when sampling thousands of markers across the genome (Manthey *et al*. 2017; Aguillon *et al.* 2018). The paradoxical conjunction of shallow genetic divergence and marked phenotypic differentiation echoes the genomic dynamics of other avian hybrid zones, namely the golden-winged *Vermivora chrysoptera* and blue-winged *Vermivora cyanoptera* complex, wherein only a few genomic regions associated with genes that determine plumage color and pattern differentiate the two species (Toews *et al.* 2016). A chromosome-level reference genome for the complex will not only facilitate the identification of the genetic basis of phenotypes (Kratochwil and Meyer 2015), a long-standing goal in evolutionary biology research, but also provide researchers a valuable resource for the examination of emerging fields in genome biology, such as the evolutionary dynamics of transposable element (TE) proliferation (Manthey *et al.* 2018), for which woodpeckers are especially well suited.

Here, we describe Caur_TTU_1.0, a *de novo* assembly that was built from a wild caught *C. auratus* female. We used three sequencing strategies: 10× Chromium, PacBio, and chromatin conformation capture (Hi-C) to assemble the first published chromosome-level genome for *C. auratus* and Picidae. As whole-genome sequencing becomes more feasible and prevalent, high-quality reference genomes will undoubtedly serve as essential resources. We expect the chromosome-level assembly presented here will be of great use to those interested in the genomic evolution of woodpeckers and birds, at large.

## Materials and methods

### DNA extraction, library preparation, and sequencing

We obtained breast muscle tissue from a vouchered *C. auratus* specimen (MSB 48083) deposited at the Museum of Southwestern Biology (MSB). The specimen was a wild female collected on July 11, 2017 in Cibola County, New Mexico (see MSB database for complete specimen details) and exhibited the ‘red-shafted’ morphology associated with *C. auratus* populations of western North America. We used a combination of 10× Chromium, PacBio, and Hi-C sequencing data for genome assembly. 10× Chromium library sequencing was carried out by the HudsonAlpha Institute for Biotechnology (Huntsville, AL, USA). They performed high-molecular weight DNA isolation, quality control, library preparation, and shotgun sequencing on one lane of an Illumina HiSeqX. For long-read PacBio sequencing, we used the services of RTL Genomics (Lubbock, TX, USA). They performed high-molecular weight DNA isolation using Qiagen (Hilden, Germany) high-molecular weight DNA extraction kits, PacBio SMRTbell library preparation, size selection using a Blue Pippin (Sage Science), and sequencing on six Pacific Biosciences Sequel SMRTcells 1M v2 with Sequencing 2.1 reagents. Hi-C library preparation was performed with an Arima Genomics Hi-C kit (San Diego, CA, USA) by the Texas A&M University Core facility. The Hi-C library was then sequenced on a partial lane of an Illumina NovaSeq S1 flow cell at the Texas Tech University Center for Biotechnology and Genomics.

### Genome assembly, polishing, scaffolding, and quality assessment

We generated an initial assembly using the raw PacBio long reads with CANU v 1.7.1 (Koren *et al.* 2017). Reads were corrected, trimmed, and assembled using CANU default parameters, while specifying a normal coarse sensitivity level (−corMhapSensitivity flag), setting the expected fraction error in an alignment of two corrected reads to 0.065 (−correctedErrorRate flag) and setting the estimated genome size to 1.6 Gb, which corresponds with previous estimates within *Colaptes* (Wright et al. 2014). We subsequently polished the PacBio assembly using the 10× Chromium sequencing reads with one iteration of the PILON v 1.22 (Walker *et al.* 2014) pipeline, which consisted of several steps. We first used bbduk, part of the BBMap v38.22 package (Bushnell 2014), to trim adapters and quality filter the raw 10× Chromium reads. We then used the BWA-MEM implementation of the Burrows-Wheeler algorithm in BWA v 0.7.17 (Li and Durbin 2010) to align these filtered reads to the PacBio assembly. We used samtools v 1.9 (Li *et al.* 2009) to sort and index the resulting BAM file, which along with the PacBio assembly, was input to PILON. Following polishing, we then performed scaffolding of the PacBio assembly with the 10× Chromium reads using ARCS (Yeo et al. 2018). An interleaved linked reads file of the 10× Chromium reads produced in LongRanger v 2.2.2 was subsequently input to the ARCS pipeline, which implements LINKS v1.8.5 (Warren *et al.* 2015). Three rounds of ARCS were performed, wherein each round multiple iterations of the pipeline were run to evaluate which parameter combination produced the assembly of highest quality. Default parameters of the pipeline were used, with the following exceptions: (1) the link ratio between two best contig pairs (-a flag), which was set to 0.5; (2) the minimum link number of links to compute scaffold (-l flag), which was set to 3; (3) the minimum sequence identify (-s flag), was varied between 97, 98, and 99; (4) the contig head/tail length for masking alignments was varied between 10k, 30k, 60k, and 100k. After all iterations were run, the assembly with greatest scaffold N50 and size was selected and used in subsequent rounds. Lastly, we used the Hi-C reads to further scaffold and fix mis-assemblies using the 3D-DNA pipeline (Durand *et al.* 2016; Dudchenko *et al.* 2017).

To assess the spatial order of the scaffolds of the Caur_TTU_1.0 assembly, we aligned it to the Chicken *Gallus gallus* chromosome-level assembly (GRCg6a, GCF_000002315.6, https://www.ncbi.nlm.nih.gov/genome/? term=Gallus%20) using the nucmer module of MUMMER v 4.0.0b2 (Kurtz *et al.* 2004). We subsequently filtered alignments using MUMMER’s delta-filter module while setting the minimum alignment identity to 70% and allowing many-to-many alignments. A tab-delimited text file that includes information on the position, percent identity, and length of each alignment was produced using MUMMER’s show-coords module (Supplementary File S18). This file was used as input to create a synteny plot with OmicCircos (Hu *et al.* 2014; R Core Team 2018). Subsequently, the Caur_TTU_1.0 scaffolds were renamed according to their corresponding Chicken chromosome. Scaffolds that did not show strong synteny to Chicken chromosomes were not renamed.

Genome assembly metrics were obtained using the function stats.sh from the BBMap v 38.22 package (Bushnell 2014). Genome completeness was estimated using Tetrapoda and Aves single-copy orthologous gene sets from both BUSCO v3 (Simão *et al.* 2015; Waterhouse et al. 2018) and BUSCO v4 (Seppey *et al.* 2019). We submitted our genome assembly to the NCBI genome submission portal, where a scan for contaminants detected no abnormalities in our assembly.

### Genome annotation

#### Repetitive element annotation and window analysis:

We annotated TEs and repetitive content in the Caur_TTU_1.0 assembly using a custom *de novo* repeat library and RepBase vertebrate database v 24.03 (Jurka *et al.* 2005). The custom repeat library was constructed from the *C. auratus* genome assembly (prior to Hi-C scaffolding) and other in-progress lab genome assembly projects in songbirds (Supplementary File S15).

Using the RepBase vertebrate database and the *de novo* repeat library, we used RepeatMasker v 1.332 (Smit *et al.* 2013–2015) to mask and summarize repetitive and TEs in the Caur_TTU_1.0 assembly (Supplementary Files S16 and S17). An interspersed repeat landscape was then produced for the Caur_TTU_1.0 assembly using the RepeatMasker scripts calcDivergenceFromAlign.pl and createRepeatLandscape.pl. The spatial distribution of repetitive content across the Caur_TTU_1.0 assembly was evaluated using custom R scripts (R Core Team 2018), first by removing overlapping elements from the RepeatMasker output, followed by a calculation of repetitive element content of the Chicken-renamed scaffolds across 100 kbp nonoverlapping sliding windows.

To generate the custom repeat library, we first input the *C. auratus* assembly that lacked Hi-C scaffolding to RepeatModeler v 1.10.11 (Smit and Hubley 2008–2015) to identify repeats *de novo*. RepeatModeler identifies repeats according to homology, repeats, and repetitiveness with the programs RECON (Bao and Eddy 2002), RepeatScout (Price *et al.* 2005), and Tandem Repeats Finder (Benson 1999). We then removed RepeatModeler sequences that were ≥98% identical to the RepBase vertebrate database. Next, we used blastn v 2.9.0 (Camacho *et al.* 2009) and bedtools v 2.29.2 (Quinlan and Hall 2010) to extract sequence matches to these novel repeats from the aforementioned assembly. We then used these sequences to create consensus sequences for each novel repetitive element using the following workflow: (1) alignment of reads using MAFFT (Katoh and Standley 2013) as implemented in Geneious (BioMatters Ltd.); (2) generation of 50% majority consensus sequences from these alignments in Geneious; and (3) trimming ambiguous nucleotides on the ends of consensus sequences. For novel repetitive elements whose ends were not recovered in the generation of the consensus sequences, we repeated the prior procedure and extracted sequences from the reference genome with 1000-bp flanks on each side of the blastn match, followed by alignment and consensus sequence generation as mentioned above (Platt *et al.* 2016). This process was repeated up to three times. We then BLASTed all novel repeats against the RepBase database to assess similarity via homology to previously characterized elements. Similarity to RepBase elements was used for naming purposes.

#### Gene annotation and window analysis:

We employed MAKER v 2.31.10 (Cantarel *et al.* 2008) to annotate putative genes in the Caur_TTU_1.0 assembly. We used the custom repeat library and protein datasets of four species in MAKER to predict genes. The species included were: (1) *Picoides pubescens* (GCF_000699005.1_ASM69900v1_Picoides_pubescens_protein.faa), (2) *Merops nubicus* (GCF_000691845.1_ASM69184v1_Merops_nubicus_protein.faa), (3) *Apaloderma vittatum* (GCF_000703405.1_ASM70340v1_Apaloderma_vittatum.protein.faa), (4) and *Buceros rhinoceros* (GCF_000710305.1_ASM71030v1_Buceros_rhinoceros_protein.faa) (Zhang *et al.* 2014). We then used these predictions to train the *ab initio* gene predictors SNAP (Korf 2004) and Augustus v.3.2.3 (Stanke *et al.* 2008). Lastly, using the SNAP and Augustus-trained gene models, we ran a second round of MAKER to annotate genes in the Caur_TTU_1.0 assembly. The spatial distribution of coding sequences (CDS) across theChicken-renamed scaffolds of the Caur_TTU_1.0 assembly was evaluated using a custom R script (R Core Team 2018).

#### Mutation rate estimation:

We extracted the putative CDS (Supplementary File S14) from the Caur_TTU_1.0 assembly using the final MAKER output and bedtools. In addition, we downloaded the CDS for *A. vittatum*, *M. nubicus*, and *B. rhinoceros* for homology-based comparisons (using the same genomes containing the aforementioned protein datasets). We performed a reciprocal BLAST of all species versus *C. auratus* using blastn to identify putative homologs across all four species (Supplementary File S19).

To put the evolution of the CDS regions in a timed evolutionary context, we downloaded a phylogenetic tree comprising all orders of Neoaves (Jarvis *et al.* 2014) and pruned the tree to the four representative orders covered by our CDS downloads and the Caur_TTU_1.0 assembly using the R package ape (Paradis *et al.* 2004): Piciformes, Coraciiformes, Trogoniformes, and Bucerotiformes.

We used T-Coffee (Notredame *et al.* 2000) to align the putative homologs between the four passerine species. T-Coffee translates nucleotide sequences, aligns them using several alignment algorithms, takes the averaged best alignment of all alignments, and back translates the protein alignments to provide a nucleotide alignment for each gene. Prior to back-translating, we removed any gaps in the protein alignments using trimAl (Capella-Gutiérrez *et al.* 2009).

With the alignments for all genes, we tested for selection using the gene-wide and branch-specific tests for selection utilized in CODEML (Yang 1997). Any alignments with gene-wide or branch-specific evidence for selection were removed for mutation rate analyses, after correcting for multiple tests using the Benjamini and Hochberg (1995) method to control false discovery rate. From each gene alignment, we used the R packages rphast, Biostrings, and seqinr (Charif and Lobry 2007; Hubisz *et al.* 2011; Pagès *et al.* 2017) to extract fourfold degenerate sites from each alignment. We concatenated the fourfold degenerate sites (*N* ∼ 528,000) and used jModelTest2 (Darriba *et al.* 2012) to determine an appropriate model of sequence evolution. We used the GTR + I model of sequence evolution in PhyML v 3.3.20190321 (Guindon and Gascuel 2003; Guindon *et al.* 2010) and user-specified tree (from Jarvis *et al.* 2014) to estimate branch lengths based on the fourfold degenerate sites. Lastly, we divided the *Colaptes*-specific branch length of this tree by the mean and 95% credible interval of the fossil-calibrated time estimate for the Piciformes-Coraciiformes divergence (also from Jarvis *et al.* 2014) to estimate a mean and 95% credible interval of potential *Colaptes-*lineage-specific mutation rates.

### Data availability

The Caur_TTU_1.0 assembly is available at NCBI (BioProject PRJNA616131; Genome JAAWVA000000000). All associated raw sequencing data, PacBio (SRR12364887), Chromium 10x (SRR12363123), and Hi-C (SRR12363461) are available from NCBI SRA. Scripts, associated files, and workflows used for this project are available on GitHub (github.com/jphruska/Colaptes_genome). Outputs from BUSCO, Maker, RepeatMasker along with the custom repeat library, a tab-delimited text file including information on mummer alignments, and a text file including information on the homologs used for mutation rate estimation are deposited as supplemental files in figshare: https://doi.org/10.25387/g3.12821822

## Results and discussion

### Sequencing, genome assembly, and synteny mapping

**Figure 1:**
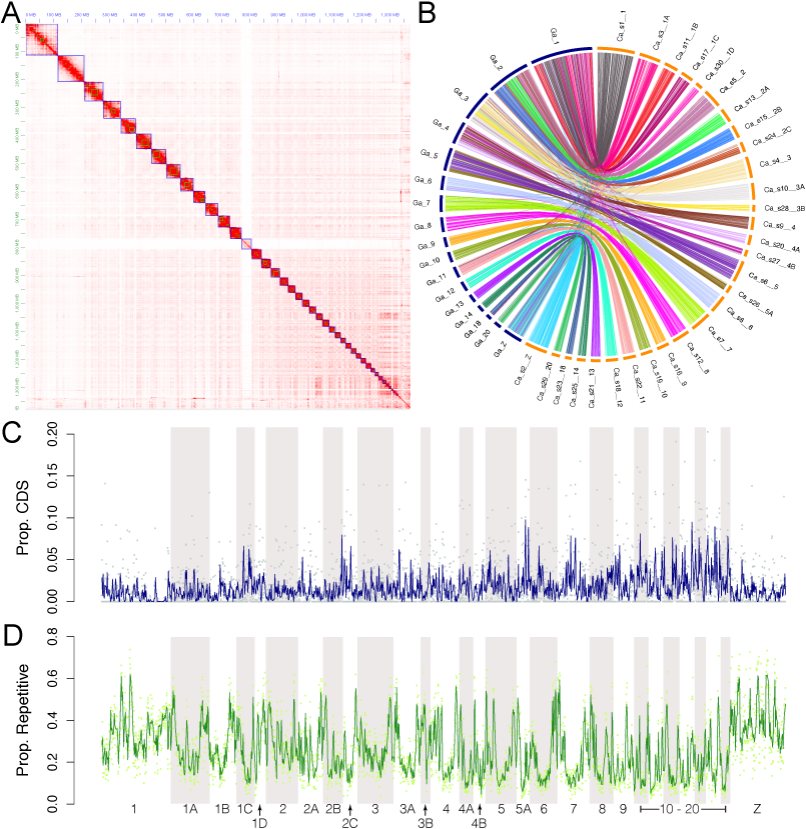
Characteristics of the Caur_TTU_1.0 assembly. (A) Hi-C scaffolding contact map. Relative contact between contigs is indicated by the intensity of red. Blue squares indicate scaffold boundaries. (B) Synteny map of Caur_TTU_1.0 (right; orange) scaffolds to *Gallus gallus* chromosomes (left; blue). (C) Proportions of CDS (top panel) and repetitive elements (bottom panel) across 100-kbp sliding nonoverlapping windows of the Chicken-aligned Caur_TTU_1.0 scaffolds. Lines indicate mean values across 10 sliding nonoverlapping windows.

Reads were generated across three sequencing approaches, including 3.94 × 10^6^ Pacific Biosciences (PacBio) long-reads (∼34× coverage), 4.47 × 10^8^ 10× Chromium paired-end reads (∼58× raw coverage), and 3.25 × 10^8^ Hi-C paired-end reads (>24,000× physical distance coverage after deduplication). The final assembly had an L50 of 43.938 Mbp scaffolds and an N50 of 11 (Figure 1A;Table 1). In terms of contiguity (L50 and N50), this assembly represents a ∼3× improvement over a recently published long-read-based Picidae assembly (*Melanerpes aurifrons* GCA_011125475.1; Wiley and Miller 2020) and represents the first published chromosome-level assembly for Piciformes. BUSCO results also suggested this assembly is of high quality, with modestly high recovery of complete bird-specific and tetrapod-specific gene groups (87.4–91.7%; Table 2; Supplementary Files S1–S12). While a higher gene group recovery rate would be expected for a highly contiguous assembly, we highlight that these results correspond with studies that have found that greater assembly contiguity often does not result in an increased gene group recovery rate, and if an increase is noted, it is often modest (Korlach *et al.* 2017; Low *et al.* 2019). Indeed, we find our recovery rates to be similar to those of the *M. aurifrons* assembly, with 92.6% of complete BUSCO gene groups recovered from the aves_odb9 dataset (Wiley and Miller 2020). We recovered a high degree of one-to-one synteny with the Chicken *Gallus gallus* chromosomes, particularly between those of small and medium size (Figure 1B). However, we note that one-to-one synteny to the *Gallus* assembly was lacking for the larger chromosomes, indicative of chromosomal splitting since the *Gallus*-*Colaptes* common ancestor has occurred. Members of Picidae are known for containing a high number of chromosomes, particularly micro-chromosomes (Kaul and Ansari 1978), and karyotypes of *Colaptes* have been shown to consistently have a larger number of chromosomes when compared to *Gallus* (Pollock and Fechheimer 1976; de Oliveira *et al.* 2017).

**Table 1:** Genome assembly metrics calculated using BBMap

Statistic	Caur_TTU_1.0
**# scaffolds / contigs**	2,369 / 9,565
**Largest scaffold / contig**	117.313 Mbp / 15.844 Mbp
**Total length**	1.378 Gbp
**Scaffold / contig N50**	11 **/** 281
**Scaffold / contig N90**	33 / 4,370
**Scaffold / contig L50**	43.948 Mbp / 826.96 Kbp
**Scaffold / contig L90**	14.604 Mbp / 50.09 Kbp
**GC (%)**	44.93

**Table 2:** BUSCO output using tetrapoda_odb9, tetrapoda_odb10, aves_od9, and aves_odb10 databases

	tetrapoda_odb9	tetrapoda_odb10	aves_odb9	aves_odb10
**Complete BUSCOs**	3,623 (91.7%)	4,670 (87.9%)	4,416 (89.9%)	7,294 (87.4%)
**Complete and single-copy BUSCOs**	3,594 (91.0%)	4,617 (86.9%)	4,342 (88.3 %)	7,224 (86.6%)
**Complete and duplicated BUSCOs**	29 (0.7%)	53 (1.0%)	74 (1.5 %)	70 (0.8%)
**Fragmented BUSCOs**	147 (3.7%)	124 (2.3%)	227 (4.6 %)	219 (2.6%)
**Missing BUSCOs**	180 (4.6 %)	516 (9.8%)	272 (5.6 %)	825 (10.0%)
**Total BUSCO groups searched**	3,950	5,310	4,915	8,338

### Genome annotation, window analysis, and mutation rate estimation

**Figure 2:**
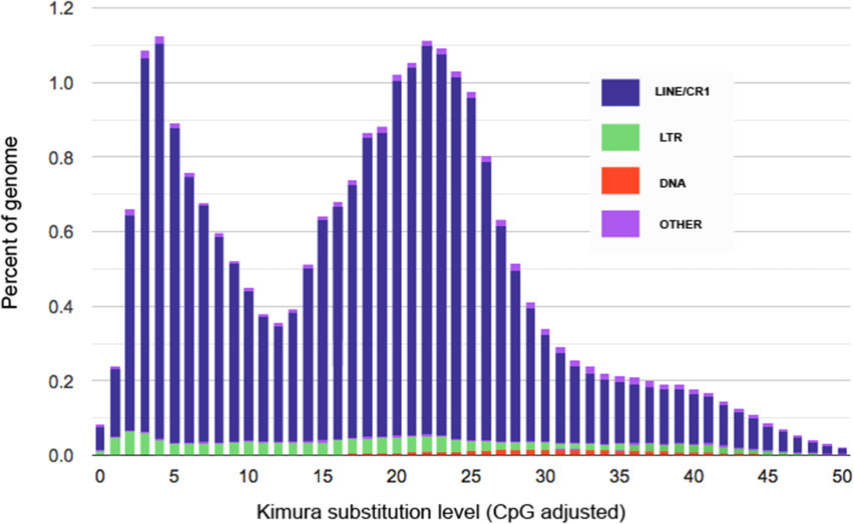
Caur_TTU_1.0 divergence landscape of TE classes. Relative abundance and age of each class are shown.

Repetitive elements make up a large portion of Caur_TTU_1.0, comprising ∼386 Mb (∼28%) of the assembly. When compared to other vertebrates, avian genomes contain comparatively low repetitive content (Sotero-Caio *et al.* 2017). The *Gallus gallus* genome, for example, comprises ∼10% repetitive elements (Hillier *et al.* 2004), which is representative of repetitive content across most lineages of birds, and is dwarfed by the 28–58% repetitive content in mammalian genomes (Platt *et al.* 2018). Piciformes, on the other hand, are somewhat of an exception, and are well-known to contain some of the highest densities of TEs in birds (Kapusta and Suh 2017), with repetitive densities often greater than 20% (*e.g.*, Zhang *et al.* 2014; Manthey *et al.* 2018; Wiley and Miller 2020). The presence of the retrotransposon superfamily CR1 (chicken repeat 1) was particularly prevalent, comprising ∼20.9% (∼287 Mbp) of the Caur_TTU_1.0 assembly. Two independent waves of CR1 proliferation were detected, with a large proportion of CR1 elements being of relatively young or medium age, as estimated by a molecular clock (Figure 2). These results echo Manthey *et al.* (2018), which also uncovered at least three waves of CR1 activity across the evolutionary history of extant Piciformes. Window analysis of repetitive elements suggested that the distribution of these elements was uneven across the assembly, both within and across scaffolds (Figure 1C). Repetitive element content was particularly prevalent near scaffold boundaries and on the Z chromosome, with local repetitive densities reaching ∼60%. High repetitive content on the Z chromosome has been reported as a pattern in woodpeckers (Bertocchi *et al.* 2018) and other birds, more generally (Kapusta and Suh 2017).

Two rounds of the MAKER annotation pipeline identified a total of 18,745 genes (mean length: 14,676.1 bp) and 149,433 exons (mean length: 161.351 bp) (Supplementary File S13). The quantity of genes and exons recovered is in line with previously annotated bird genomes (Zhang *et al.* 2014). The distribution of CDS across 100-kbp sliding windows of the Caur_TTU_1.0 assembly revealed that, as expected, these sequences comprised a smaller fraction of autosomal and sex chromosomes when compared to repetitive elements (Figure 1C).

The mutation rate analysis of fourfold degenerate sites from neutrally evolving genes suggests that the mean rate in *C. auratus* is 4.007 × 10^−9^ substitutions/site/year; with a 95% credible interval = 3.525 × 10^−9–^4.976 × 10^−9^. This rate is ∼1.5× higher than a previous estimate of the Downy Woodpecker *Dryobates pubescens* (2.42 × 10^−9^; Nadachowska-Brzyska *et al.* 2015). While these results could be reflecting biologically distinct mutation rates between species of woodpeckers, we also acknowledge this discrepancy in results could result from differing methodological choices. Therefore, we urge caution when interpreting this result.
